# IFN-gamma signaling in the central nervous system controls the course of experimental autoimmune encephalomyelitis independently of the localization and composition of inflammatory foci

**DOI:** 10.1186/1742-2094-9-7

**Published:** 2012-01-16

**Authors:** Eunyoung Lee, Sarah Chanamara, David Pleasure, Athena M Soulika

**Affiliations:** 1 Institute for Pediatric Regenerative Medicine, Shriners Hospitals for Children Northern California, Sacramento, California, USA, 95817; 2 Department of Dermatology, School of Medicine, University of California, Davis Sacramento, California, USA, 95816; 3 The Department of Neurology, School of Medicine, University of California Davis, Sacramento, California, USA, 95817

**Keywords:** microglia, cerebellum, brainstem, EAE, IFNγ, STAT1, inflammation

## Abstract

**Background:**

Murine experimental autoimmune encephalomyelitis (EAE), a model for multiple sclerosis, presents typically as ascending paralysis. However, in mice in which interferon-gamma (IFNγ) signaling is disrupted by genetic deletion, limb paralysis is accompanied by atypical deficits, including head tilt, postural imbalance, and circling, consistent with cerebellar/vestibular dysfunction. This was previously attributed to intense cerebellar and brainstem infiltration by peripheral immune cells and formation of neutrophil-rich foci within the CNS. However, the exact mechanism by which IFNγ signaling prohibits the development of vestibular deficits, and whether the distribution and composition of inflammatory foci within the CNS affects the course of atypical EAE remains elusive.

**Methods:**

We induced EAE in IFNγ-/- mice and bone marrow chimeric mice in which IFNγR is not expressed in the CNS but is intact in the periphery (IFNγR^CNS^KO) and vice versa (IFNγR^peri^KO). Blood-brain barrier permeability was determined by Evans blue intravenous administration at disease onset. Populations of immune cell subsets in the periphery and the CNS were quantified by flow cytometry. CNS tissues isolated at various time points after EAE induction, were analyzed by immunohistochemistry for composition of inflammatory foci and patterns of axonal degeneration.

**Results:**

Incidence and severity of atypical EAE were more pronounced in IFNγR^CNS^KO as compared to IFNγR^peri^KO mice. Contrary to what we anticipated, cerebella/brainstems of IFNγR^CNS^KO mice were only minimally infiltrated, while the same areas of IFNγR^peri^KO mice were extensively populated by peripheral immune cells. Furthermore, the CNS of IFNγR^peri^KO mice was characterized by persistent neutrophil-rich foci as compared to IFNγR^CNS^KO. Immunohistochemical analysis of the CNS of IFNγ-/- and IFNγR chimeric mice revealed that IFNγ protective actions are exerted through microglial STAT1.

**Conclusions:**

Alterations in distribution and composition of CNS inflammatory foci are not sufficient for the onset of atypical EAE. IFNγ dictates the course of neuroinflammatory disorders mainly through actions exerted within the CNS. This study provides strong evidence that link microglial STAT1 inactivation to vestibular dysfunction.

## Introduction

Experimental autoimmune encephalomyelitis (EAE) is the most commonly used animal model for multiple sclerosis (MS). Similar to MS, animals with EAE exhibit activation of immune cells in peripheral immune organs, migration of these cells into the CNS, and establishment of multifocal inflammation, demyelination and damage to neurons and axons. Cumulative axonal loss eventually leads to severe and permanent neurological deficits [1,2]. In classical EAE, most neurological deficits are attributable to spinal cord lesions. Interestingly, mice in which IFNγ signaling has been genetically disrupted (IFNγ-/-, or IFNγR-/- mice), also show evidences of cerebellar/vestibular dysfunction, deficits that are regarded as atypical EAE features. Such deficits in MS patients are associated with poor prognosis [3-5]. This atypical EAE course is behaviorally characterized by deficits such as axial rotation, circling behavior, and balance impairment. In this study we sought to determine the mechanisms by which interrupted IFNγ signaling leads to this atypical course.

IFNγ is a multifunctional cytokine that is involved in the initiation and establishment of inflammation, and participates in both innate and adaptive immune responses. T and NK cells are the main sources of IFNγ [6,7] but it can be induced in many other cell types including neurons [8,9]. IFNγ is also crucial for the resolution of inflammation by inducing apoptosis of activated lymphocytes, supporting Treg function, and restricting the development of T cell subsets associated with autoimmunity [10,11]. Thus, the exacerbation of neurological disability in animals with EAE in the absence of IFNγ or its receptor has been attributed in the past to uncontrollable expansion of pathogenic immune cells in the periphery [12-14].

However, IFNγ has yet-to-be fully defined actions on neurons, astrocytes, oligodendrocytes and microglia, all of which express IFNγR [8,15-20]. It is possible that IFNγ acts as a disease-limiting agent within the CNS but not in peripheral immune tissues. This would explain why peripherally administered IFNγ has no effect on the course of EAE, while intraventricularly administered IFNγ alleviates or resolves the neurological deficits [21-23].

Alternatively, the protective effect of IFNγ signaling in EAE may be exerted both in peripheral immune cells and within the CNS [22,24,25]. Passively transferred EAE is characterized by increased cerebellar/brainstem infiltration and atypical neurological deficits when IFNγ signaling is disrupted in either the recipient mouse or the donor cells [24]. One drawback of passively transferred EAE, however, is that immune cells from the recipient animal are also activated and migrate into the CNS parenchyma along with those of the donor [26,27], thus making it challenging to dissociate peripheral from CNS events. Furthermore, passively transferred IFNγ-/- cells will also perturb IFNγ induced signaling in CNS resident cells; this is because, in EAE, infiltrating immune cells are the major IFNγ source in the CNS. For these reasons, it remains unclear whether the development of atypical deficits in the absence of IFNγ is due to effects on peripheral immune cells before or after they enter the CNS, or directly on CNS resident cells.

Finally, although in EAE mice without IFNγ signaling, infiltration of the cerebellum or brainstem by peripheral immune cells has been considered sufficient for the development of atypical deficits, EAE-susceptible mouse strains with intact IFNγ signaling show infiltration of these areas but never develop atypical deficits [28-30].

In this study, we re-visited the role(s) of IFNγ signaling in the genesis of atypical EAE. We initially characterized the disease process in IFNγ-/- mice. Then, to differentiate between peripheral and CNS-restricted roles of IFNγ signaling, we constructed irradiation bone marrow chimeric mice in which IFNγR was expressed in radioresistant CNS cells but not in radiosensitive peripheral immune cells or vice versa.

Our data shows that active EAE induction in mice lacking IFNγR in the CNS results in more frequent and more severe atypical neurological deficits compared to mice lacking IFNγR in peripheral immune cells. This difference in incidence of atypical EAE was not attributable to more extensive cerebellar infiltration, preferential recruitment of pathogenic immune cell subsets within the CNS, or increased axonopathy. Instead, our data are consistent with the hypothesis that IFNγ exerts protective actions against vestibular/brainstem/cerebellar dysfunction in EAE via activation of the microglial IFNγR-STAT1 pathway.

## Methods

### Mice

IFNγ-/- and IFNγRKO, C57BL/6 (C57BL/6CD45.2) mice and B6.SJL-*Ptprc^a ^Pepc^b^*/BoyJ (C57BL/6CD45.1) were purchased from Jackson Laboratory. Both males and females (matched per experiment) were used. Mice were housed in a pathogen-free facility. All experimental protocols were approved by the Institutional Animal Care and Use Committee of the University of California, Davis.

### Generation of bone marrow chimeras (BMC)

Recipient mice (6 weeks old) were irradiated with 950 rads (Cs-137 source, MK1-30 irradiator, J.L Shepherd and Assoc.) 18 to 24 hours before bone marrow transfer, and treated with antibiotic water (sulfamethoxazole, Hi-Tech Pharmacal) for two weeks thereafter. Bone marrow cells were harvested from femurs and tibias of donor mice and red blood cells were lysed with ACK solution (Quality Biological) for 3 min at 37°C. T cells were removed by complement mediated lysis, by resuspending bone marrow cells at a concentration of 10^7 ^cells/mL in Cytotoxic media, RPMI-1640 containing 25 mM Hepes buffer and 0.3% bovine serum albumin, followed by successive incubation with Thy1.2 antibody (1:500, CEDARLANE) at 4°C for 30 min and Low Tox M rabbit complement (1:10, CEDARLANE) at 37°C for 30 min. Cells were resuspended in PBS and counted. Ten million bone marrow cells in 150 μl were injected into the recipient mice via tail vein injection the day after lethal irradiation. EAE was induced 6 to 8 weeks post bone marrow reconstitution. BMC constructed are described in Table 1.

**Table 1:** Irradiation bone marrow chimeric mice (BMC)

Donor mice	Recipient mice	BMC mice
IFNγRKO-CD45.2	IFNγRKO-CD45.2	IFNγRKO-chimeras

C57BL/6-CD45.1	IFNγRKO-CD45.2	IFNγR^CNS^KO

C57BL/6-CD45.1	C57BL/6-CD45.2	WT-chimeras

IFNγRKO-CD45.2	C57BL/6-CD45.1	IFNγR^peri^KO

Radiosensitive (peripheral immune) cells carry the genotype of donor mouse, while radioresistant cells (all parenchymal cells including the CNS) carry the genotype of the recipient mouse.

### EAE induction

EAE was induced as previously described [1]. Briefly, 300 μg of rodent MOG peptide (amino acids 35-55, New England Peptides) in CFA containing 5 mg/ml killed Mycobacterium tuberculosis (Difco) were administered subcutaneously in the flank to age-matched (11-14 weeks-old) mice on day 0. Non-irradiated mice received 200 ng of pertussis toxin and bone marrow chimeras 50 ng of pertussis toxin intraperitoneally on days 0 and 2. Both classical and atypical neurological deficits were evaluated daily until day 35 post immunization (pi). Classic neurological deficits were graded as follows: limp tail or waddling gait = 1; limp tail and waddling gait = 2; single limb paresis and ataxia 2.5; double limb paresis = 3; single limb paralysis and paresis of second limb = 3.5; full paralysis of 2 limbs = 4; moribund = 4.5; and death = 5 [1,31]. Atypical neurological deficits were graded as previously described [32] with slight modifications: Mild head tilting = 1, severe head tilting = 2, body tilting = 3, involuntary and continuous axial rotation = 4.

### Mapping areas of blood brain barrier breakdown by Evans blue

Mice were intravenously injected with 200 μl of 2.5% Evans blue (Sigma) in saline. Ninety minutes later, mice were anesthetized by intraperitoneal injection of ketamine (150 mg/kg) and xylazine (16 mg/kg) and intracardially perfused with ice cold PBS, followed by 4% paraformaldehyde (PFA) in PBS. The brains and spinal cords were harvested, post-fixed in 4% PFA overnight at 4°C. The next day, tissues were sectioned at sites of Evans blue staining and photographed using the Zeiss SteREO Lumar.V12.

### Isolation of leukocytes from mouse spleen/lymph nodes and CNS

Mice sacrificed by CO_2 _asphyxiation were perfused with ice cold PBS. Spleens and draining lymph nodes were harvested, combined, minced in PBS, and pushed with the back of a syringe plunger through a 40 μm mesh. Red blood cells were lysed with ACK solution (Quality Biologicals). Brains and spinal cords were minced, digested at 37°C for 30 min in PBS containing 0.04 units of Liberase R1 (Roche) and 10 μg of DNase I (Roche) per ml. Softened fragments were pushed through a 100 μm mesh. CNS infiltrating cells were isolated via a discontinuous 40/70% (v/v) Percoll gradient (GE Healthcare).

### *Ex vivo *T cell responses

Mixed splenocytes and lymph node cells were cultured in 200 μl of RPMI 1640 containing 10% FBS, 2 mM L-glutamine, 0.1 mM nonessential amino acids, 100 U penicillin-streptomycin, 50 μM 2-mercaptoethanol, and 1 mM sodium pyruvate with or without 50 μg/ml MOG peptide (amino acids 35-55) for 24 hrs. The cells were incubated with brefeldin A (GolgiPlug, BD Bioscience) for the last 5 hr.

### Flow cytometry

Mixed splenocytes and lymph node cells were immunostained immediately after isolation or after the 24 hour culture described above. CNS mononuclear cells were immunostained after incubation at 37°C for 3 hours in RPMI 1640 containing 10% FBS, 2 mM L-glutamine, 0.1 mM nonessential amino acids, 100 U/mL penicillin-streptomycin, 50 μM 2-mercaptoethanol, and 1 mM sodium pyruvate in the presence of brefeldin A. Immediately prior to immunostaining, Fc receptors were blocked for 10 min with anti-CD16/32. Neutrophils were identified by Fluorescein Isothiocyanate (FITC)-labeled anti-mouse Ly-6G and Pe-Cy™7-labeled anti-mouse Gr1 (BD pharmingen), macrophages or microglia by Phycoerythrin (PE)-labeled anti-mouse CD45, BD pharmingen and eFlour^® ^450-labeled anti-mouse CD11b (eBioscience), and dendritic cells by Allophycocyanin (APC)-labeled anti-mouse CD11c (BD Pharmingen). To distinguish donor cells from recipient cells, cells were co-stained with FITC labeled anti-mouse CD45.2 (BD pharmingen) and APC-labeled anti-mouse CD45.1 (eBioscience). For T helper (Th) and T cytotoxic (Tc) cell subset analysis, cells were stained with Pacific Blue (PB)-labeled anti-mouse CD4 and FITC-labeled anti-mouse CD8a (BD pharmingen), fixed, permeabilized using the Cytofix/Cytoperm Plus Kit (BD pharmingen) according to the manufacturer's protocol, and intracellularly stained with APC-labeled anti-mouse IFNγ, PE-labeled anti-mouse IL17 and Pe-Cy™7-labeled anti-mouse TNFα (BD pharmingen). Th1: CD4+IFNγ+, Th17: CD4+IL17+, Th1/17: CD4+IFNγ+IL17+ Tc1: CD8+IFNγ+, Tc17: CD8+IL17+, Tc1/17: CD8+IFNγ+IL17+. For regulatory T lymphocyte (Treg, CD4+CD25+Foxp3+) analysis, cells were stained with PB-labeled anti-mouse CD4 and APC-labeled anti-mouse CD25 (eBioscience) with Fc receptor blocker, fixed, and permeabilized using the Fixation & Permiabilization kit (eBioscience), then intracellularly stained with PE-labeled anti-mouse/rat Foxp3 (eBioscience). Immunostained cells were analyzed using a Cyan FACS (Dako Cytomation).

### Immunohistology of spinal cord and cerebellum/brainstem

Mice were anesthetized and brains and spinal cords tissues were harvested as described in the "*Mapping areas of blood brain barrier breakdown by Evans blue" *section. Harvested tissues were fixed overnight in 4% PFA, immersed in 30% sucrose for 48 hours at 4°C and embedded in cryostat mounting media (Tissue-Tek OCT, Sakura Finetek). Tissues were coronally or sagittally sectioned at 10 μm thickness. Sections were air-dried at RT for 30 min, rinsed briefly with PBS, and blocked with 10% of donkey or goat serum, depending on the secondary antibody used. Some sections were blocked with antibody diluent (IHC-Tek™). Sections were incubated with primary antibodies listed in Table 2 overnight at 4°C and next day, with fluorescently conjugated secondary antibodies to DyLight™488 or DyLight™549 (Jackson ImmunoResearch) for an hour at RT. Between each step, the sections were washed repeatedly with PBS for 5 min period to reduce background. Nuclei were visualized by DAPI counterstain. Fluorescent images were analyzed by confocal microscopy (Nikon A1). Microscopic fields were photographed with 20× or 40× objectives mounted on a Nikon A1 scanning confocal microscope using the Nikon NIS-Elements AR 3.10 software. Exposure was adjusted per staining for all groups of mice using Adobe Photoshop.

**Table 2:** Primary antibodies used for immunohistochemistry

Antigen	Antibody
Ly6G	FITC-conjugated rat anti-mouse Ly6G BD Pharmingen (Cat#551460), 1:100

IBA1	Rabbit polyclonal anti-IBA1 Wako (Cat#019-19741), 1:1000

CD11b	Rat anti-mouse CD11b BD Pharmingen (Cat#550282), 1:100

CD4	Rat anti-mouse CD4 BD Pharmingen (Cat# 550280), 1:100

CD45.1	PE-conjugated mouse anti-mouse CD45.1 eBioscience (Cat#12-0453-82), 1:100

CD45.2	PE-conjugated mouse anti-mouse CD45.2 BD Pharmingen (Cat# 560695), 1:100

CD45.2	FITC-conjugated mouse anti-mouse CD45.2 eBioscience (Cat#11-0454-85)

SMI32	Mouse anti-mouse NF-H Covance (Cat# SMI-32R), 1:1000

STAT1	Rabbit polyclonal anti-human STAT1 Santa Cruz (Cat# SC592), 1:100

CD45R	PE-Texas Red rat anti-mouse CD45R/B220 BD Pharmingen (Cat# 551489), 1:100

IL4	Goat anti-mouse IL4 R&D (Cat# AF-404-NA)

MBP	Rat anti-bovine myelin basic protein Novus biologicals (Cat# NB600-717)

#### Quantification of SMI32+ axons, demyelinating foci, CD45R+ cells or IL4+ cells

Fields encompassing the whole spinal cord or cerebellum were photographed using a 20× objective mounted on a Nikon laser scanning confocal microscope, and images were tiled together using the Nikon NIS-Elements software. All SMI32+ axons in the white matter of spinal cords and cerebella isolated from mice with EAE were counted with the aid of NIH ImageJ software. Demyelinating foci (absence of MBP staining) were traced in the white matter of spinal cords and cerebella isolated from mice with EAE were measured with the aid NIS-Elements software. Total white matter areas were measured in the cross-sections of cerebella or spinal cords and were used for normalization in the quantification of SMI32+ axons and demyelinating foci. Total spinal cord or cerebellum cross-section areas were used for normalization when quantifying CD45R+ or IL4+ cells. Three 10 μm sections (40-50 μm apart from each other) were counted per animal, n = 4 animals. To determine statistical significance between the chimeric groups a Kruskal-Wallis test with post-hoc Mann-Whitney U tests with Bonferroni correction was performed.

### Numbers of mice used in the study

#### Flow cytometry

WT and IFNγ-/-: To calculate significance, p-values were derived using the Mann-Whitney U test with Bonferroni correction. The experiment was repeated 2-3 times, employing 3 mice per experiment for a total of 6-9 mice per time-point per group.

IFNγRKO chimeras: To determine statistical significance between the chimeric groups a Kruskal-Wallis test with post-hoc Mann-Whitney U tests with Bonferroni correction was performed. The experiment was repeated 3 times employing 2 mice per experiment for a total of 6 mice per time-point per group.

#### Immunohistochemistry

The experiment was repeated 2-3 times, employing 2-3 mice per experiment for a total of 4-9 mice per time-point per group.

## Results

### Atypical neurological deficits initiate in the absence of IFNγ signaling in the CNS

#### Clinical course in wild type and IFNγ -/- mice

**Figure 1:**
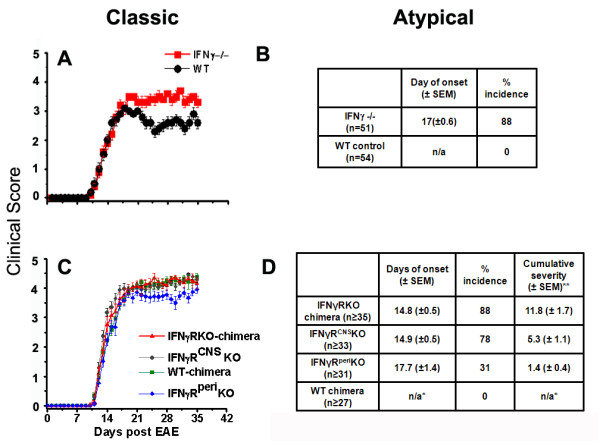
**EAE course in IFNγ-/- and WT mice and IFNγR-KO chimeras**. Classic (A and C) and atypical deficits (B and D) were monitored and documented daily as described in the methods. Data are represented as means ± SEMs (A and B, n=51-54, C and D, n=13-20). *n/a: not applicable, ** Average cumulative atypical score of mice with EAE up to day 20 post-immunization.

Classic neurological deficits of EAE (ascending paralysis) appeared both in IFNγ-/- and wild-type (WT) controls on or around day 10 post-immunization (pi). During the chronic phase of EAE (post day 21), WT mice improved slightly, but IFNγ-/- mice continued to display severe typical symptoms (Figure 1A). Atypical symptoms such as head tilting, continuous axial rotation, and leaning towards one side developed only in IFNγ-/- mice, starting on or a few days after the onset of the classic EAE course (Figure 1B).

#### Clinical course in IFNγRKO chimeras

Classic neurological deficits appeared in all irradiation bone marrow chimeric groups on or around day 11 pi. IFNγRKO-chimeras, IFNγR^CNS^KO mice, and WT-chimeras showed classic deficits of similar severity throughout the course of their illness, while IFNγR^peri^KO mice displayed slightly milder disease severity during the chronic phase of EAE (Figure 1C). Atypical neurological deficits developed in 88% of IFNγRKO-chimeras on day 14.8 ± 0.5 (means ± SEM) but were not present in WT-chimeras (Figure 1D). Interestingly, EAE induction in mice without IFNγ signaling in the CNS (IFNγR^CNS^KO) resulted in the development of more severe and more frequent atypical disease, that had an earlier onset than mice without IFNγ signaling in the periphery (IFNγR^peri^KO): 78% of IFNγR^CNS^KO (day of onset 14.9 ± 0.5, and average cumulative score 5.3 ± 1.1) versus 31% of IFNγR^peri^KO (day of onset 17.7 ± 1.4, and average cumulative score 1.4 ± 0.4) developed atypical EAE (Figure 1D).

### Peripheral immune responses and CNS leukocyte accumulation are not correlated with the incidence of atypical symptoms

To detect possible associations between atypical disease and activation of peripheral immune cells and/or preferential recruitment of immune subsets into the CNS, we isolated cells from lymphoid organs and from Percoll-fractionated pooled brain and spinal cord of the various groups of mice at the peak of disease (days 14-21 pi) and analyzed them by flow cytometry as described in the methods.

#### IFNγ-/-/WT

**Figure 2:**
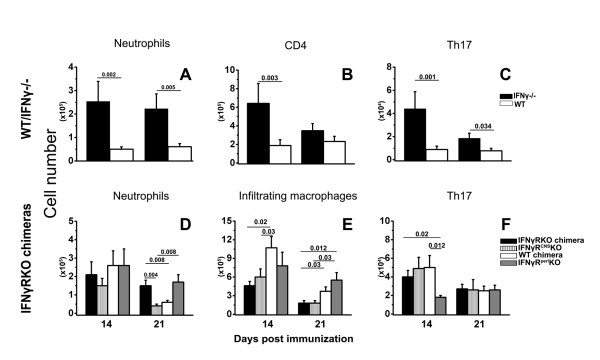
**Peripheral immune responses and CNS leukocyte accumulation are not correlated with the incidence of atypical symptoms**. Cell subsets isolated from Percoll-fractionated CNS (pooled spinal cord and brain) showing significant changes by flow cytometry between the various groups of mice with EAE. Neutrophils (A) and Th17 cells (C) were significantly upregulated on days 14 and 21 pi, and total CD4+ T cells on day 14 (B) in the CNS of IFNγ-/- compared to WT controls. The CNS of IFNγRKO-chimeras and IFNγR^peri^KO mice, on day 21 (but not day 14) pi was populated by greater numbers of neutrophils (Ly6G+GR1+) compared to WT-chimeras and IFNγR^CNS^KO mice (D). Infiltrating macrophages were significantly upregulated only in the CNS of WT-chimeras (CD11b+CD45.1^hi^Ly6G-) and IFNγR^peri^KO mice (CD11b+CD45.2^hi^Ly6G-) both on day 14 and 21 pi (E). Fewer Th17 cells were detected in the CNS of IFNγR^peri^KO mice compared to the other groups only on day 14 pi (F). Data are presented as means ± SEMs, n = 6-9; significance was calculated using the Mann-Whitney test with Bonferroni correction for the WT and IFNγ-/- groups and with the non-parametric Kruskal-Wallis with post-hoc Mann-Whitney U test with Bonferroni correction for the IFNγRKO- chimeric groups.

Significantly increased numbers of peripheral MOG-specific Th17 cells were generated (Additional file 1) in IFNγ-/- mice compared to WT controls only on day 14 pi (p = 0.001). As expected, CD4+IFNγ+ (Th1) CD4+IFNγ+IL17+ (Th1/17), CD8+IFNγ+ (Tc1) and CD8+IFNγ+IL17+ (Tc1/17) cells were only observed in WT controls and not in IFNγ-/- mice. No other statistically significant differences between the two groups were observed in the numbers of immune cell subsets in peripheral lymphoid organs (Additional file 1). In the CNS, the numbers of neutrophils, total CD4+ T and Th17 cells were significantly increased in IFNγ-/- mice as compared to WT controls (Figure 2 A-C). These observations suggest that global absence of IFNγ facilitates the accumulation of neutrophils and CD4+ T cells in the CNS, in agreement with previous studies [12,24,33]. Furthermore, the increased numbers of Th17 cells in the CNS of IFNγ-/- mice may imply preferential migration and/or (re)polarization of these cells within the CNS; however, the decreased ratios of Tregs:Th17 in the CNS of IFNγ-/- mice as compared to WT mice (p = 0.007, Additional file 1) imply that in the absence of IFNγ, Tregs may not efficiently control the Th17-induced neurotoxicity [34].

#### IFNγRKO chimeras

To determine the degree of chimerism, we used CD45.1/CD45.2 allelic markers to identify host versus donor infiltrating cells. All of the chimeric mice exhibited 85-95% of donor cells in the periphery. Mice from each chimeric group were randomly selected, sacrificed 6-8 weeks post reconstitution and mononuclear cells were isolated from the CNS. We found that in the CNS of chimeric mice, microglia were typically 85-95% of recipient origin (data not shown). We were not able to collect such data for IFNγRKO-chimeric mice since both the recipient and donor cells carry the CD45.2 allele.

There were no significant differences between the four groups of chimeric mice in the peripheral immune cell subsets examined on days 14 and 21 pi. However, by day 21, IFNγRKO chimeric mice tended to exhibit more TNFα-producing cells in the periphery as compared to the three other groups. In the CNS, the total numbers of CD4+ and CD8+ T cells and microglia of the various groups of chimeric mice were also comparable (Additional file 1). As in WT-mice, WT-chimeras and IFNγR^peri^KO mice tended to exhibit higher Treg:Th17 ratios compared to IFNγRKO-chimeras and IFNγR^CNS^KO mice; however these differences did not reach statistical significance. In addition, in the CNS of the IFNγR^peri^KO mice CD8+ T cells were elevated but this again did not reach statistical significance (Additional file 1).

Nevertheless, the differential expression of IFNγR did influence the composition of the infiltrating immune cells. On day 14, IFNγR^peri^KO mice had statistically significantly lower numbers of Th17 in the CNS compared to the rest of the chimeric groups (Figure 2F), which was not due to defective Th17 cell generation in the periphery of these mice (Additional file 1). However, by day 21 all groups exhibited similar numbers of Th17 cells within the CNS.

Although there was no difference between the chimeric groups in the numbers of neutrophils (Ly6G+GR1+) within the CNS at disease onset (day 14 pi), a week later (day 21 pi), these numbers were significantly higher in IFNγRKO-chimeras and IFNγR^peri^KO mice compared to WT-chimeras and IFNγR^CNS^KO mice (Figure 2D). Thus, the migration of neutrophils into the CNS was IFNγ-independent, but their maintenance in (or continuous migration to) the CNS was facilitated by the absence of IFNγ signaling in peripheral immune cells.

B cells have been implicated in the generation of atypical EAE [35]. B cells were quantified by immunohistochemistry using an anti-CD45R antibody. As shown in additional file 1, on day 14, B cells numbers were not statistically significantly different between the chimeric groups. On day 21 however, B cells number were statistically significantly decreased in IFNγR^CNS^KO and increased in IFNγR^peri^KO compared to WT-chimeras and IFNγRKO-chimeras.

Expression of IFNγR in the CNS, independent of its expression in the periphery, enhanced CNS accumulation of infiltrating macrophages [WT-chimeras (CD11b+CD45.1^hi^Ly6G-) and IFNγR^peri^KO (CD11b+CD45.2^hi^Ly6G-)] both on days 14 and 21 (Figure 2E), suggesting that macrophage migration cues are regulated by IFNγ-induced events within the CNS (e.g. chemokine expression by CNS resident cells).

Furthermore, IFNγR^CNS^KO mice showed a similar composition of infiltrating cells (neutrophils, T cell subsets) to WT-chimeras. These two groups exhibited differences in infiltrating macrophages but only on day 14: WT-chimeras showed increased numbers of infiltrating macrophages compared to both IFNγR^CNS^KO and IFNγRKO-chimeras (Figure 2). On day 21, increased numbers of macrophages observed in WT-chimeras were statistically significantly different than those in IFNγRKO chimeras but did not reach statistical significance compared to those in IFNγR^CNS^KO mice. Macrophages showed the same trends in WT-chimeras and IFNγR^peri^KO mice.

However, the composition of the infiltrates did not directly affect the incidence of atypical neurological deficits, since severe atypical deficits developed in both IFNγR^CNS^KO and IFNγRKO chimeras, though the makeup of CNS infiltrating cells differed dramatically between these two mouse groups.

These observations suggest that the composition of the CNS-infiltrating cells is determined by IFNγ-induced events taking place both in the periphery and the CNS, but do not solely control the onset of atypical disease.

### The spatial pattern of CNS infiltration is determined by IFNγ signaling in peripheral immune cells, but does not correlate with the incidence of atypical EAE

**Figure 3:**
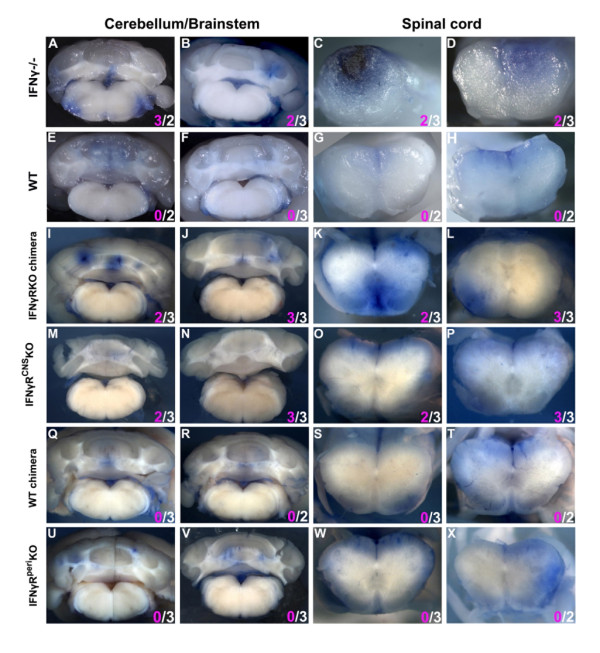
**BBB permeability does not correlate with onset of atypical disease**. Representative Evans blue infiltrated areas of the cerebella/brainstems and spinal cords of the various groups of mice at EAE onset. CNS areas showing Evans blue staining (see Additional file 2) were cut transversely and photographed using a Zeiss stereoscope. Two (out of 4-6) representative mice per group are shown. IFNγ-/- (A-D), IFNγRKO-chimeras (I-L) and IFNγR^peri^KO (U-X) exhibited substantial Evans blue staining both in cerebellum and spinal cord. Evans blue staining patterns in the WT (E-H), WT-chimeras (Q-T) and IFNγR^CNS^KO (M-P) were meningeal and confined in small areas in the spinal cord and were rarely detected in the cerebellum/brainstem parenchyma. Numbers in pink and white denote atypical and classic neurological deficits, respectively.

Previous reports suggested that prominent cerebellar and/or brainstem infiltration is sufficient to initiate atypical symptoms in IFNγ-/- mice with EAE [26,32]. To evaluate this assumption, we compared the degree of infiltration of the cerebella/brainstems of mice displaying atypical deficits with that of mice that developed typical deficits only, after EAE induction. To determine the areas of blood-brain barrier permeability, mice were injected intravenously with Evans blue (Eb) within 24 hours after the onset of neurological deficits; 90 min later, mice were perfused intracardially with PBS and brains and spinal cords were analyzed as described in the methods. All chimeric mice exhibited Eb staining in similar areas of the CNS (Figure 3 and Additional file 2). However, the depth of parenchymal Eb infiltration varied substantially between the groups. The CNS of mice with global absence of IFNγ signaling (IFNγ-/- and IFNγRKO chimeras) showed intense Eb intra-parenchymal staining, sometimes reaching the gray matter (Figure 3 A-D and 3I-L) both in the cerebellum/brainstem and spinal cord. Similar Eb staining patterns were observed in the CNS of IFNγR^peri^KO mice (Figure 3 U-X), extending deeply and broadly into the parenchyma. Notably, IFNγR^peri^KO mice showed intense Eb staining in the cerebellum and brainstem.

On the contrary, Eb+ foci in the CNS of WT mice, WT-chimeras and IFNγR^CNS^KO mice were usually confined to the meninges or minimally penetrating the glia limitans into the superficial parenchyma (Figure 3E-H and 3M-T). Furthermore, Eb staining in the parenchyma of the cerebellum/brainstem of IFNγR^CNS^KO mice was rarely observed.

As noted above, IFNγR^CNS^KO mice displayed more frequent and more severe atypical deficits than IFNγR^peri^KO mice. Thus the intense Eb staining in the cerebella/brainstem of IFNγR^peri^KO mice compared to IFNγR^CNS^KO mice indicates that extensive infiltration of these areas is not sufficient to elicit atypical EAE.

### Composition of inflammatory foci within the CNS does not affect the onset of atypical neurological deficits

Next, we tested the hypothesis that differential expression of IFNγR causes preferential accumulation of distinct immune cell subsets in specific CNS areas (e.g. cerebellum versus spinal cord). We, thus, isolated cerebellar/brainstem, and spinal cord tissue from mice with EAE and examined the composition of their inflammatory foci by immunohistochemistry.

**Figure 4:**
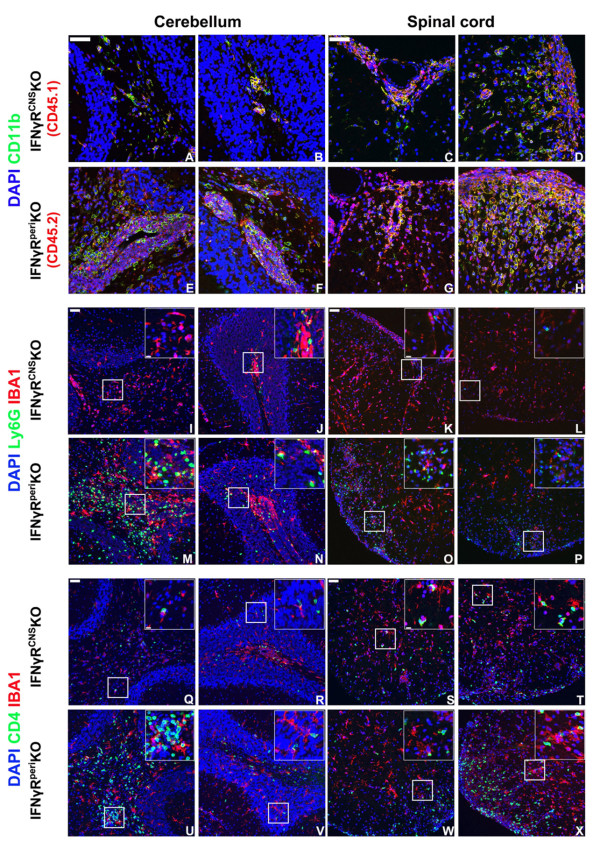
**Composition of inflammatory foci in the CNS of mice with EAE does not correlate with onset of atypical disease**. Mice were sacrificed on day 21 pi and CNS infiltration was examined by immunohistochemistry. In IFNγR^CNS^KO mice CD11b+CD45.1+ infiltrating cells were usually found perivascularly or close to the meninges (A-D). In IFNγR^peri^KO mice, CD11b+CD45.2+ infiltrating cells were found both in perivasular spaces and deep into the parenchyma (E-H). There were few Ly6G+ neutrophils in the cerebella and spinal cords of IFNγR^CNS^KO mice (I-L) while the same areas of IFNγR^peri^KO mice were characterized by prominent neutrophilic infiltration (M-P). IFNγR^CNS^KO exhibited decreased CD4+ T cell parenchymal infiltration (Q-T) compared to IFNγR^peri^KO (U-X). Space bar = 50 μm. Inserts show magnified fields demonstrating close interactions between IBA1+ and Ly6G + (I-P) or CD4+ (Q-X) cells (space bar = 10 μm). Nuclei were visualized by DAPI staining. Two (out of 4-6) representative mice per set are shown.

We used CD11b as a generic marker of inflammatory foci. CD11b is easily detectable by immunohistochemistry on infiltrating macrophages, dendritic cells and neurtophils along with NK and some CD8+ T cells [36-40]. Microglia strongly express CD11b when activated but do so only faintly when in resting state. We also used the CD45 allelic markers CD45.1 and CD45.2 to discriminate between infiltrating cells and microglia in the chimeric mice. Infiltrating CD11b+CD45.1+ cells in IFNγR^CNS^KO mice and WT-chimeras cells remained clustered and close to the vasculature and meninges (Figure 4 A-D and Additional file 3). In the CNS of IFNγR^peri^KO mice and IFNγRKO chimeras, infiltrating CD11b+CD45.2+ cells were abundant and widely distributed throughout the CNS parenchyma (Figure 4 E-H and Additional file 3). Neutrophils (Ly6G+) were abundant in the CNS of all chimeric groups on day 14 (not shown). On day 21, however, neutrophils were rarely detected in the CNS of IFNγR^CNS^KO mice (Figure 4 I-L) or WT mice and WT-chimeras (Additional file 4), but remained prominent in the CNS lesions of IFNγR^peri^KO mice (Figure 4 M-P) and of IFNγ-/- mice and IFNγRKO-chimeras (Additional file 4). Furthermore, CD4+ T cells were more often found in perivascular and meningeal spaces and close to the glial limitans, and had only barely penetrated into the parenchyma in the CNS of IFNγR^CNS^KO (Figure 4 Q-T) WT mice and WT-chimeras (Additional file 5). On the contrary, CD4+ T cells were usually found deep in the parenchyma in the CNS of IFNγR^peri^KO (Figure 4 U-X), IFNγ-/- and IFNγRKO chimeras (Additional file 5),

Anti-IBA1 antibody labels more intensely activated and quiescent microglia, and less intensely, infiltrating macrophages. IBA1+ cells migrated to the site of infiltration and were in close contact with neutrophils and CD4+ T cells (Figure 4), probably attempting to control tissue damage. By staining for the CD45 allele markers CD45.1/CD45.2, we verified that the cells interacting with infiltrating CD4+T and Ly6G+ cells are of recipient origin (i.e: microglia, Additional file 6).

In summary and in agreement with the Eb patterns, these data show that infiltrates in IFNγ-/-, IFNγRKO-chimeras and IFNγR^peri^KO mice penetrated deeply into the parenchyma and were widely scattered, whereas inflammatory foci in WT mice, WT-chimeras and IFNγR^CNS^KO mice were constrained to the perivascular spaces and the meninges and tightly clustered together. Lesion composition was similar in all mice, consisting of CD4+T, CD11b+ and IBA1+ cells. The main difference in the inflammatory foci between the groups of mice was the persistence of Ly6G+ neutrophils in IFNγ-/- IFNγRKO chimeras and IFNγR^peri^KO a week after disease onset (day 21), at which time point they were sparsely observed in WT mice, WT-chimeras and IFNγR^CNS^KO mice. These data, in agreement with our flow cytometry results, indicate that the intra-lesion composition is not directly associated with the onset of atypical EAE. However, intense intercellular interactions between microglia and infiltrating cells may play deciding roles on the actions of peripheral cells after these enter the CNS parenchyma.

### The patterns of axonal degeneration are not linked to the development of atypical symptoms

Cumulative axonal injury is largely responsible for the development of permanent neurological disability in MS patients and animals with EAE [1,2,41,42]. We speculated that in the absence of IFNγ signaling in the CNS, axonal damage would be more severe, in the cerebellum and brainstem (areas that control posture), or in areas of the spinal cord, as unilateral damage of the spinal cord has been associated with balance impairments, such as leaning towards one side and disequilibrium [43]. To detect axonal damage by immunohistochemistry, we used the SMI32 antibody, which is specific for hypophosphorylated neurofilament H (hypo-NF-H). Hypo-NF-H resides only in neuronal bodies of the gray matter of the healthy spinal cord. In the healthy CNS, SMI32 immunoreactivity is not detected in the white matter (Additional file 7). SMI32 immunoreactivity, within the white matter is indicative of accumulation of hypo-NF-H in axons, reflecting axonal flow disturbances, degeneration or transection [1,44].

**Figure 5:**
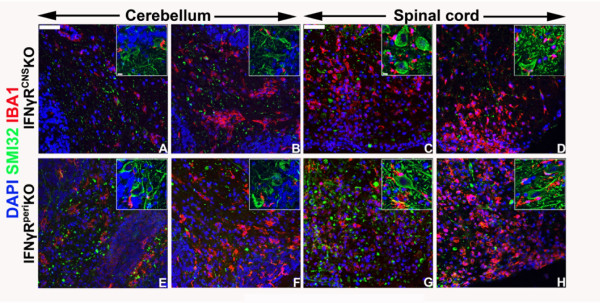
**Axonal damage, as visualized by SMI32+ axons in the white matter, correlates with inflammatory foci but not with atypical EAE**. Cerebella and spinal cords isolated on day 21 pi were analyzed for axonal damage by immunoreactivity for SMI32 antibody. SMI32+ axons were detectable in the white matter only within or around inflammatory foci. SMI32 immunoreactivity was less intense in cerebella and spinal cords of IFNγR^CNS^KO (A-D) compared to IFNγR^peri^KO (E-H). Space bar = 50 μm. Inserts show magnified areas of the gray matter depicting microglia (IBA1+) in close contact with neuronal bodies (SMI32+). Space bar = 10 μm. Nuclei were visualized by DAPI staining. Two (out of 4-6) representative mice per set are shown.

The pattern of SMI32 immunoreactivity in the white matter correlated with the degree of infiltration. Axonal damage occurred only within and around inflammatory foci. Thus, IFNγ-/- mice, IFNγRKO-chimeras and IFNγR^peri^KO mice showed intense SMI32 immunoreactivity both in the cerebellum and spinal cord compared to WT mice, WT-chimeras IFNγR^CNS^KO mice (Figure 5, Additional file 7). However, quantification of SMI32 immunoreactive axons within the white matter of chimeric mice with EAE did not show statistically significant differences between the groups (Additional file 7).

Quantification of demyelinating foci in the same sections visualized by absence of myelin basic protein (MBP) showed no difference between the groups in the spinal cord. Interestingly, however, the cerebellum of IFNγR^CNS^KO mice with EAE exhibited smaller and fewer areas of demyelinating foci (Additional File 8), although this did not reach statistical significance (p = 0.0522).

These data indicate that the pattern of axonal damage as detected by accumulation of hypo-NF-H in the white matter could not account for the onset of atypical neurological deficits. However, in the CNS of all bone marrow chimeric mice with EAE, IBA1+ microglia were found in close contact with neuronal perikarya and axons (Figure 5 and Additional file 7), suggesting that these intercellular communications affect neuronal function.

### Atypical neurological deficits develop preferentially in the absence of microglial IFNγR-STAT1 signaling

#### STAT1 is upregulated in the CNS only in response to IFNγ

**Figure 6:**
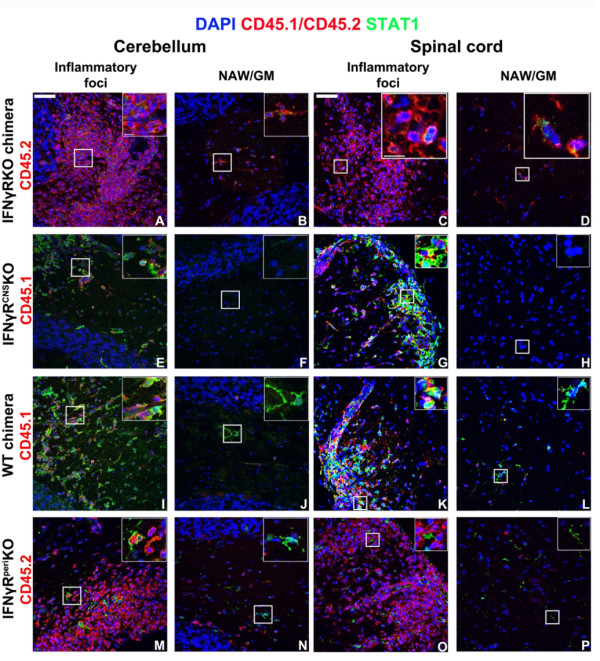
**STAT1 expression is detected mainly in IFNγR+ cells**. Panels show areas of inflammatory foci or normal appearing white/gray matter (NAW/GM) areas away from the site of inflammation. Infiltrating cells are shown in red (CD45.2 for A-D and M-P or CD45.1 for E-L). IFNγRKO-chimeras expressed CD45.2 both in the CNS and periphery, and STAT1 was rarely detected in isolated cells within infiltrating foci (A, C) or in the normal appearing white/gray matter (NAW/GM) areas away from the site of inflammation (B, D). IFNγR^CNS^KO mice exhibited STAT1 expression in CD45.1+(IFNγR+) infiltrating cells (E, G) and not in the NAW/GM (F, H). WT-chimeras expressed STAT1 both in CD45.1+ (IFNγR+) infiltrating cells and CD45.1-(IFNγR+) resident cells (I-L), while in IFNγR^peri^KO mice, STAT1 expression was mainly absent from CD45.2+(IFNγR-) infiltrating cells (M, O) but was localized in CD45.2-(IFNγR+) resident cells in NAW/GM (N, P). Space bar = 50 μm. Inserts show selected magnified fields. Space bar = 10 μm. Nuclei were visualized by DAPI staining. Two (out of 4-6) representative mice per set are shown.

In the absence of reliable antibodies against IFNγR for immunohistochemistry, to identify the cell type(s) that respond to IFNγ signaling we examined the expression patterns of the transcription factor STAT1 that is downstream from IFNγR. STAT1 was not detected in the healthy CNS (Additional file 9). To confirm that STAT1 is upregulated only in cells that express IFNγR, we initially examined STAT1 expression by co-labeling for CD45.1 or CD45.2. In IFNγRKO-chimeras, both microglia and infiltrating cells carried the CD45.2 allele. STAT1 was rarely detected and only within inflammatory foci (Figure 6 A-D). On the contrary, WT-chimeras with EAE showed abundant STAT1 expression in both infiltrating (CD45.1+) and resident cells (CD45.1-) (Figure 6 I-L). In the CNS of IFNγR^CNS^KO mice with EAE, infiltrating CD45.1+ (IFNγR+), but not resident CD45.1- (IFNγR-) cells, expressed STAT1 (Figure 6 E-H). In IFNγR^peri^KO mice, STAT1 was expressed by resident CD45.2- (IFNγR+) but not infiltrating CD45.2+ (IFNγR-) cells (Figure 6 M-P). These observations indicated that in the inflamed CNS, STAT1 is specifically upregulated in response to IFNγ signaling.

#### IFNγ signals mainly in CD11b+ cells in the inflamed CNS

**Figure 7:**
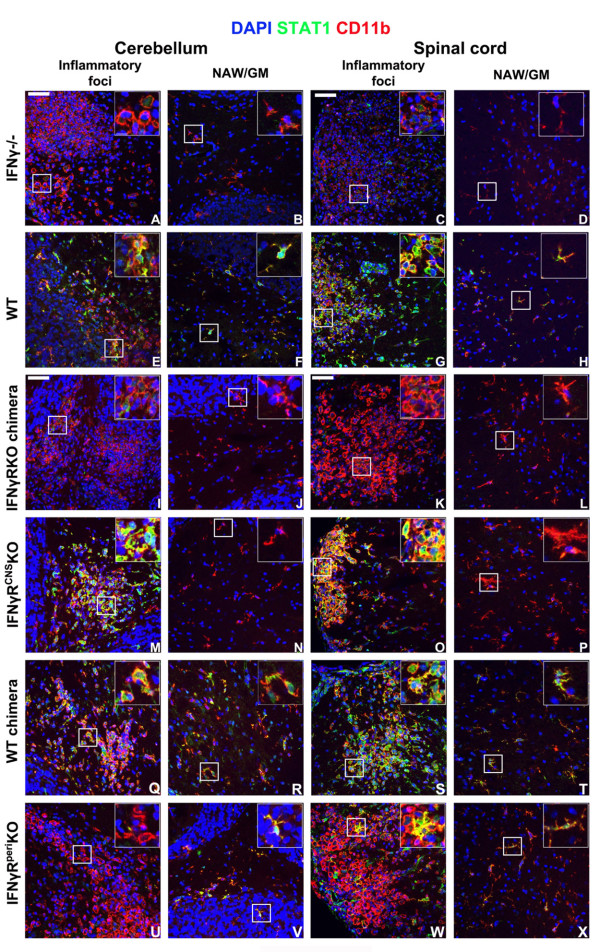
**STAT1 is mainly expressed in CD11b+ cells**. Cerebella and spinal cords isolated from the different groups of mice on day 14 post EAE induction were analyzed for STAT1 expression close to the inflammatory foci and in the normal appearing white or gray matter (NAW/GM). In the CNS of IFNγ-/- mice and IFNγRKO-chimeras STAT1 expression was rarely detected, while CD11b+ cells were abundant both in the cerebellum and spinal cord (A-D, I-L). STAT1 expression was readily detectable in the CNS of WT mice and WT-chimeras both within the inflammatory foci (E, G, Q and S) and in the NAW/GM (F, H, R and T). In IFNγR^CNS^KO mice, STAT1 expression was prominent in CD11b+ cells within the inflammatory foci (M, O), while it was rarely detected in CD11b+ cells with ramified morphology away from the inflammatory lesions (N, P). In the CNS of IFNγR^peri^KO mice, the majority of CD11b+ cells with amoeboid morphology (activated microglia or macrophages) in the inflammatory foci did not express STAT1 (U, W). STAT1 was mainly expressed by CD11b+ cells located in the normal appearing gray matter, exhibiting morphology similar to that of ramified microglia (V, X). Space bar = 50 μm. Inserts show selected magnified fields to confirm STAT1 upregulation. Space bar = 10 μm. Nuclei were visualized by DAPI staining. Two (out of 4-6) representative mice per set are shown.

Further examination revealed that STAT1 was upregulated mainly in CD11b+ cells with intact IFNγ signaling (Figure 7). STAT1 was almost absent in the CNS parenchyma of IFNγ-/- and IFNγRKO chimeras with EAE, although CD11b immunoreactivity was abundant (Figure 7 A-D and 7I-L). In WT mice and WT-chimeras with EAE, STAT1 immunoreactivity was abundant in CD11b+ cells in inflammatory foci representing infiltrating cells and activated microglia, both in the cerebellum and the spinal cord (Figure 7 E, G and 7Q, S). Furthermore CD11b+ cells with ramified morphology further from the site of inflammation in the otherwise normal appearing white and gray matter were also STAT1 positive (Figure 7 F, H and 7R, T). This suggests that microglia respond to IFNγ signaling even when they are not in close contact with infiltrating cells. In IFNγR^CNS^KO mice, STAT1 was upregulated in CD11b+ cells within the inflammatory foci (IFNγR+) but not in CD11b+ resident cells (IFNγR-) (Figure 7 M-P). In IFNγR^peri^KO mice, STAT1 expression was prevalent in CD11b+ cells with microglial morphology (resident cells, IFNγR+) and not in infiltrating cells (IFNγR-) in the white matter (Figure 7 U-X). Occasionally, STAT1 expression was detected in GFAP+ cells (astrocytes) found close to inflammatory foci, but only in the presence of intact IFNγ signaling within the CNS (Additional file 10). Furthermore, STAT1 was usually found within the nucleus, suggesting activation of the STAT1 trancription process (Additional File 11).

These data clearly show that, IFNγ-elicited STAT1 responses in the inflamed CNS are largely restricted to CD11b+ cells. Absence of STAT1 in infiltrating CD11b+ cells does not correlate to a large extent with the onset of atypical EAE (IFNγR^peri^KO mice). On the contrary, atypical neurological deficits are strongly associated with inactivation of the IFNγ-STAT1 signaling pathway in microglia (IFNγR^CNS^KO mice).

## Discussion

In the past, the development of atypical EAE in mice without IFNγ signaling has been attributed to preferential infiltration of the cerebellum and/or brainstem by activated immune cells, increased generation of Th17 cells in the periphery, and/or formation of neutrophil-rich posterior fossa inflammatory foci [24,45-47]. To a large extent, these conclusions were derived from studies of adoptively transferred EAE, a paradigm that does not allow for clear discrimination of immune cells derived from the donor and host. Therefore, to discriminate between the actions of IFNγ in the CNS and the periphery, we constructed irradiation bone marrow chimeric mice lacking IFNγR in CNS cells but not peripheral immune cells or vice versa. After EAE induction, mice without IFNγR in radioresistant CNS cells (IFNγR^CNS^KO) had more severe and more frequent atypical neurological deficits as compared to mice lacking IFNγR in radiosensitive peripheral immune cells (IFNγR^peri^KO). Histological and flow cytometry analysis showed that lesion distribution, inflammatory cell populations within the CNS and intra-lesion composition of the inflammatory foci did not correlate with the incidence of atypical EAE. In contrast to what we had anticipated, cerebellar/brainstem areas of IFNγR^CNS^KO mice exhibited low levels of infiltration while the same areas of IFNγR^peri^KO mice were heavily populated by peripheral immune cells. Furthermore, the numbers of Th17 cells and neutrophils in the CNS did not correlate with the presence or absence of atypical EAE. In conjunction with the previous studies mentioned above, our observations indicate that although cerebellar/brainstem infiltrates of neutrophils and Th17 cells may be necessary to cause atypical EAE, they are not sufficient alone to do so. This led us to the conclusion that the CNS resident cell responses in the absence of IFNγ signaling may be responsible for the onset of atypical EAE.

We used STAT1 as a surrogate marker to identify cell targets of IFNγ signaling. STAT1 was detected mainly on CD11b+ cells, both in the inflammatory foci and in normal appearing white and gray matter. Although we can not exclude STAT1-independent events, these data show that IFNγ actions in the CNS are exerted in microglia and infiltrating CD11b+ cells. Furthermore, IFNγR^CNS^KO mice with severe atypical EAE showed virtually no STAT1 expression in microglia, but STAT1 upregulation was detected in infiltrating CD11b+ cells. On the contrary, STAT1 was not detected in infiltrating CD11b+ cells, but was widely expressed in microglia in IFNγR^peri^KO mice, which showed mild or no atypical deficits. These data suggest that deletion of IFNγ signaling in microglia, but not in infiltrating CD11b+ cells, correlates with atypical EAE. This conclusion is further strengthened by the prior observation that mice without STAT1 suffer a fulminant form of EAE [46].

Microglia (IBA1+ cells that were of host origin as verified by immunohistochemistry for the appropriate CD45 allele) were closely apposed to infiltrating CD4+ and Ly6G+ cells in the CNS of EAE mice, and thus may have modulated the properties of these peripheral immune cells after their entry into the CNS [48,49].

Activated microglia were also in close contact with neuronal perikarya and axons in the EAE mice. Upon activation, microglia may either promote neurogenesis, and support neuronal function [50-52], or accelerate or induce damage [53-55]. It is likely that in the absence of IFNγ signaling microglia become neurotoxic causing damage in spinocerebellar, cerebrospinal and propriospinal tracts that control balance and posture; these tracts transverse both the brain stem and the spinal cord. Damaged axons are most abundant in and around inflammatory foci in EAE, and are likely to contribute to motor deficits such as ascending paralysis [1]. However, the extent of axonal damage as detected by SMI32 immunoreactivity in the cerebellum and spinal cord was not correlated with the incidence of atypical EAE, though it is possible that axonal and neuronal damage detectable by techniques other than SMI32 immunostaining (e.g., alterations in mitochondrial morphology [56], dysregulated Na+ channel expression [57], or disruption of neuronal microtubule network [44]) may prove to be associated with atypical neurological deficits.

Our data suggest that, in the inflamed CNS, IFNγR-STAT1 signaling is one of the factors that determines whether microglia acquire a neuroprotective or neurotoxic properties. Though the extent of neuronal death was minimal and not different in the cerebella and spinal cords of the various chimeric mice as assessed by TUNEL (data not shown) we suspect that IFNγR deficient microglial responses induce physiological alterations in the properties of specific subsets of cerebellar/brainstem neurons that lead to circling [58] and other atypical neurological symptoms.

## Conclusions

In the present study, we demonstrated that in EAE the development of deficits associated with vestibular dysfunction is not dependent solely on cerebellar and brainstem infiltration by peripheral immune cells, or the composition and localization of inflammatory foci within the CNS. Our data support the hypothesis that inhibition of IFNγ actions mediated through microglial STAT1 is the main direct (cross-talk with neurons) or indirect (cross-talk with infiltrating cells, or astrocytes) cause for the onset of atypical neurological deficits. Modulation of IFNγ-STAT1 responses in microglia might prove a promising therapy for cerebellar/vestibular dysfunction in neuroinflammatory disorders.

## Abbreviations

BMC: bone marrow chimera; CNS: central nervous system; EAE: experimental autoimmune encephalomyelitis; Eb: Evans blue; Hypo-NF-H: hypophosporylated neurofilament heavy; IFNγ: interferon-gamma; IFNγR: IFNγ receptor; pi: post immunization; KO: knockout; NK: natural killer cells; STAT1: signal transducer and activator of transcription 1; Th: T helper cells; Tregs: regulatory CD4+ T cells; WT: wild type

## Competing interests

The authors declare that they have no competing interests.

## Authors' contributions

EL performed all the experiments, analyzed data, prepared figures and help in the writing of the manuscript, SC assisted EL in collecting flow cytometry and immunohistochemistry data, DP provided help with the interpretation of axonal degeneration data, read and thoroughly reviewed the manuscript, AMS was responsible and oversaw the whole study, analyzed data and wrote the manuscript.

All authors have read and approved the final manuscript.

## Supplementary Material

Additional file 1 **Table 1: Immune cell subsets in periphery (MOG-recall) and the CNS of IFNγ-/- and WT mice with EAE. P values were calculated using Mann-Whitney U test with Bonferroni correction**. Six to nine mice were analyzed per time point in each group. Table 2: Immune cell subsets in periphery and the CNS of the chimeric mouse groups with EAE. P values were calculated using Kruskal-Wallis test with post-hoc Mann-Whitney U tests with Bonferroni correction. Six mice were analyzed per group in each time point. Click here for file

Additional file 2 **Macroscopic photographs showing BBB permeability**. Macroscopic photographs of spinal cords and brains isolated up to 24 hrs post the onset of neurological deficits and 90 minutes after Evans blue intravenous administration. Tissues were photographed using the Zeiss SteREO Lumar.V12 stereoscope and tiled together using Photoshop. Click here for file

Additional file 3 **CD11b+ cells distribution in the inflamed CNS**. CD11b+ cells were abundant in the cerebella and spinal cords of IFNγRKO-chimeras and WT chimeras on day 21 pi. CD11b+CD45.2+ cells were found within the CNS parenchyma of IFNγRKO-chimeric mice (both infiltrating and resident microglia express CD45.2 in this chimeric group, A-D). In the CNS of WT-chimeras, infiltrating CD45.1+CD11b+ cells (E-H) were usually found perivascularly or close to the meninges. Nuclei were visualized by DAPI staining. Two (out of 4-6) representative mice per set are shown. Space bar = 50 μm. Click here for file

Additional file 4 **Neutrophil distribution in the inflamed CNS**. On day 21 pi, cerebella and spinal cords of IFNγ-/- mice were heavily populated by Ly6G+ neutrophils (A-D), while the same areas of WT mice exhibited low level and spatially confined neutrophilic infiltration (E- H). Ly6G+ neutrophils were abundant in the cerebella and spinal cords of IFNγRKO-chimeras (I-L) but only sparsely detected in WT-chimeras (M-P). Nuclei were visualized by DAPI staining. Space bar = 50 μm. Inserts show magnified fields demonstrating close interactions between IBA1+ and Ly6G+ cells (space bar = 10 μm). Two (out of 4-6) representative mice per set are shown. Click here for file

Additional file 5 **CD11b+ cells distribution in the inflamed CNS**. Cerebella and spinal cords of IFNγ-/- (A-D) and IFNγRKO-chimeras (I-L) exhibited increased parenchymal infiltration by CD4+T cells compared to WT (E-H), and WT-chimeras (M-P), respectively. Two (out of 4-6) representative mice per set are shown. Nuclei were visualized by DAPI staining. Space bar = 50 μm. Inserts show magnified fields demonstrating close interactions between IBA1+ and CD4+ cells (space bar = 10 μm). Click here for file

Additional file 6 **Resident microglia interact with infiltrating cells**. Resident microglia cells identified by CD45.2 (pseudo green) for IFNγR^CNS^KO (A, B, G, H) and WT-chimeras (C, D, I, J) or CD45.1(pseudo green) IFNγR^peri^KO (E, F, K, L) closely interact with CD4+ (A-F) and Ly6G+ (G-L) infiltrating cells (pseudo red). Two (out of 4-6) representative mice per set are shown. Nuclei were visualized by DAPI staining. Space bar = 50 μm. Click here for file

Additional file 7 **Axonal damage as visualized by SMI32+ axons in the white matter does not correlate with atypical EAE**. Spinal cords and cerebella isolated from healthy mice and EAE mice on day 21 pi were analyzed for axonal damage by immunoreactivity for hypophosphorylated neurofilament-H in the white matter, using the SMI32 antibody. SMI32+ axons were not detected in the healthy white matter (A-D). SMI32 immunoreactivity is readily detected in healthy neuronal bodies within the gray matter (inserts in panels A-D). SMI32+ axons were detectable in the white matter only within or around the inflammatory foci and were more intense in the cerebella and spinal cords of IFNγ-/- mice (E-H), and IFNγRKO-chimeras (M-P) compared to WT mice (I-L), and WT-chimeras (Q-T). IBA1+ cells were found in close proximity to SMI32+ axons in the white matter in all groups of mice. Nuclei were visualized by DAPI staining. Two (out of 4-6) representative mice per set are shown. Space bar = 50 μm. Inserts show magnified areas of the gray matter depicting IBA1+ cells in contact with neuronal bodies. Space bar = 10 μm. Panel U shows the quantification of SMI32+ axons per mm^2 ^of white matter of spinal cords or cerebella isolated from the chimeric mouse groups with EAE on day 21. Click here for file

Additional file 8 **Loss of myelin does not correlate with the onset of atypical neurological dificits**. Fields encompassing the whole spinal cord or cerebellum, isolated from chimeric groups with EAE on day 21 and stained with anti-MBP antibody, were photographed using a 20X objective mounted on a Nikon laser scanning confocal microscope, and images were tiled together using the Nikon NIS-Elements. MBP negative areas were traced and quantified using the ImageJ software. Panels A-H show representative cerebellar and spinal cord sections stained with MBP; areas of demyelination are traced in white and white matter is traced in green. Panel I shows the quantification of MBP-negative areas as mm^2^/mm^2 ^of white matter. On day 14, IFNγR^CNS^KO mice showed fewer and smaller demyelinating foci compare to the other groups and this difference approached statistical significance (p = 0.0522). No other statistically significant difference was observed between the groups. Click here for file

Additional file 9 **STAT1 was not detected in healthy CNS of non-irradiated (A-D) or chimeric (E-H) mice**. CD11b immunoreactivity was not detected in healthy CNS, since microglia are not activated. Nuclei were visualized by DAPI staining. Space bar = 50 μm. Click here for file

Additional file 10 **STAT1 is expressed only in few GFAP+ astrocytes usually close to inflammatory foci**. STAT1 was not detected in GFAP+ astrocytes in IFNγ-/- (A-D) or IFNγRKO chimeras (I-L). In the CNS of IFNγR^CNS^KO (M-P) and IFNγR^peri^KO (U-X), GFAP+ cells rarely expressed STAT1, but were routinely found in close contact with STAT1 expressing cells. STAT1 was occasionally detected in astrocytes close to a lesion in the CNS of WT mice (E-H) and WT-chimeras (Q-T). Nuclei were visualized by DAPI staining. Two (out of 4-6) representative mice per set are shown. Space bar = 50 μm. Inserts show magnified fields. Space bar = 10 μm. Click here for file

Additional file 11 **STAT1 translocates to the nucleus**. Sections isolated from the cerebellum and spinal cord of chimeric mice with EAE were stained with anti-STAT1 (green) and anti-CD11b (red). The sections were imaged using a 60× objective mounted on a Nikon laser scanning confocal microscope. Panels A, B, C and D are stacked images of 14 sequential planes each 0.7 μm thick. Panels A' B' C' and D' are single plane orthogonal confocal images showing STAT1 translocation into the nucleus. Nuclei were visualized by DAPI staining. Space bar = 50 μm. Click here for file
