# Erratum to: IFN-gamma signaling in the central nervous system controls the course of experimental autoimmune encephalomyelitis independently of the localization and composition of inflammatory foci

**DOI:** 10.1186/s12974-016-0702-8

**Published:** 2016-08-30

**Authors:** Eunyoung Lee, Sarah Chamanara, David Pleasure, Athena M. Soulika

**Affiliations:** 1 Institute for Pediatric Regenerative Medicine, Shriners Hospitals for Children Northern California, Sacramento, CA 95817 USA; 2 Department of Dermatology, School of Medicine, University of California, Davis Sacramento, CA 95816 USA; 3 The Department of Neurology, School of Medicine, University of California Davis, Sacramento, CA 95817 USA

## Erratum

Upon publication of the original article [1], it was noticed that Sarah Chamanara’s name was incorrectly written as ‘Sarah Chanamara’. This has now been corrected in this erratum.
