# Supplementary material for: IFN-gamma signaling in the central nervous system controls the course of experimental autoimmune encephalomyelitis independently of the localization and composition of inflammatory foci
Source: J Neuroinflammation. 2012 Jan 16;9:7. doi: 10.1186/1742-2094-9-7 (PMC3293042; doi:10.1186/1742-2094-9-7)
Supplement: Additional file 1 — Table 1: Immune cell subsets in periphery (MOG-recall) and the CNS of IFNγ-/- and WT mice with EAE. P values were calculated using Mann-Whitney U test with Bonferroni correction. Six to nine mice were analyzed per time point in each group. Table 2: Immune cell subsets in periphery and the CNS of the chimeric mouse groups with EAE. P values were calculated using Kruskal-Wallis test with post-hoc Mann-Whitney U tests with Bonferroni correction. Six mice were analyzed per group in each time point. [file 1742-2094-9-7-S1.RTF]

.   
Additional table 1: Immune cell subsets in periphery (MOG-recall) and the CNS of IFNg-/- and WT mice with EAE. See material and methods. P values were calculated using Mann-Whitney U test with Bonferroni correction. Six to nine mice were analyzed per time point per group
							
	 	 	day 14 pi	day 21 pi	
 	Group	Cell type	Absolute number	p	Absolute number	p	
Periphery (MOG recall)*	IFNg-/-	Total cell number  (X107)	15.2 (±  1.7)	ns	  9.2 (± 1.5 )	ns	
	wt 		15.3 (±  3.0)		14.1  (±  2.8)		
	IFNg-/-	CD4+ (X107)	 2.7 (±  0.3)	ns	2.0(± 0.2 )	ns	
	wt 		 2.4 (± 0.4 )		2.9 (± 0.5)		
	IFNg-/-	  Th1(X103)	6.6 (±  2.6)	0.0003	6.2 (± 1.9)	0.0003	
	wt 		436.7 (±  104.8)		148.4             (± 45.1 )		
	IFNg-/-	  Th17 (X105)	8.3 (± 1.2)	0.001	1.9 (± 0.5)	ns	
	wt 		2.2 (± 0.5) 		1.8 (± 0.5)		
	IFNg-/-	  Th1/17 	0 (±  0)	0.0001	  0(±  0)	0.0001	
	wt 		64284 (± 13993)		31683            (± 9202 )		
	IFNg-/-	Treg (X106)	 1.8 (±  0.3)	ns	3.2 (± 0.6)	ns	
	wt 		 1.7 (±  0.4)		3.9 (±  0.9)		
	IFNg-/-	  CD8 (X107)	1.7 (± 0.2)	ns	1.2 (± 0.2)	ns	
	wt 		1.9 (± 0.5)		1.9 (± 0.5)		
	IFNg-/-	  Tc1 (X103)	  1.9 (± 0.6)	0.001	 3.2 (± 1.4)	0.002	
	wt 		  83.3(± 22.9)		  29.4 (± 12.0)		
	IFNg-/-	  Tc17 (X103)	 59.9 (± 14.7)	0.016	  5.4 (±  0.7)	ns	
	wt 		  17.9 (± 2.9)		 10.2  (±  3.3)		
	IFNg-/-	  Tc1/17 (X102)	  11.4 (±  5.9)	ns	6.8 (±  4.6)	0.031	
	wt 		  81.4 (± 11.9)		19.7 (± 5.1)		
	IFNg-/-	TNFa+ cells (X106)	 12.3 (± 2.8)	ns	 4.8 (± 1.2)	ns	
	wt 		  24.7 (± 8.6)		10.2 (± 3.5)		
* numbers obtained after 18hr culture in the presence of MOG35-55


 Additional Table 1 (continued)
	 	 	day 14 pi	day 21 pi	
 	Group	Cell type	Absolute number	p	Absolute number	p	
CNS	IFNg-/-	Total cell number  (X106)	2.1 (± 0.3)	ns	1.6 (± 0.4)	ns	
	wt 		 1.4 (± 0.4)		1.3 (± 0.3)		
	IFNg-/-	  Th1 (X102)	 1.0 (± 0.4)	0.006	1.2 (± 0.4)	0.003	
	wt 		39.6 (± 19.2)		41.0 (± 12.3)		
	IFNg-/-	  Th1/17 	46 (± 22)	0.052	8 (±  8)	0.001	
	wt 		479 (± 261)		434 (±165)		
	IFNg-/-	  Treg (X104)	3.7 (± 1.0)	ns	4.7 (± 1.0)	ns	
	wt 		2.0 (± 0.7)		5.1 (± 1.2)		
	IFNg-/-	Treg/Th17	0.7 (± 0.1)	0.003	10.4 (± 5.4)	ns	
	wt 		1.9 (± 0.3)		100.6 (± 52.3)		
	IFNg-/-	  CD8 (X104)	3.4 (± 0.5)	ns	3.2 (± 0.8)	ns	
	wt 		3.4 (± 0.9)		5.2 (± 1.3)		
	IFNg-/-	 Tc1 	30 (± 16)	0.028	4 (± 4)	ns	
	wt 		113 (± 39)		25 (± 15)		
	IFNg-/-	  Tc17 (X102)	1.7 (± 0.5)	ns	3.2 (±  1.6)	ns	
	wt 		1.2 (± 0.5)		1.0 (± 0.5)		
	IFNg-/-	  Tc1/17  (x10)	25 (± 14)	ns	4 (± 4)	ns	
	wt 		40 (± 16)		22 (± 22)		
	IFNg-/-	TNFa+ cells (X105)	2.0 (± 0.3)	ns	1.4 (± 0.3)	ns	
	wt 		2.3 (± 0.6)		1.6 (± 0.4)		
	IFNg-/-	Microglia  (X105)	5.5 (± 1.1)	ns	5.1 (± 1.3 )	ns	
	wt 		4.5 (± 1.0)		6.4 (±  1.7)		
	IFNg-/-	Macrophages (X105)	7.2 (± 1.5)	ns	3.4 (±  0.8)	ns	
	wt 		5.4 (± 1.8)		4.3 (±  1.1)		
	IFNg-/-	Dendritic cells (X105)	2.6 (± 0.4)	ns	1.5 (±  0.4)	ns	
	wt 		3.0 (± 1.0)		3.3 (± 0.9)		


Additional table 2: Immune cell subsets in periphery and the CNS of the chimeric mouse groups with EAE. P values were calculated using Kruskal-Wallis test with post-hoc Mann-Whitney U tests with Bonferroni correction. Six mice were analyzed per group per time point
		 	 	day 14 pi	day 21 pi	
Periphery	 	Group	Cell type	Absolute number	p	Absolute number	p	
	 	IFNgRKO chimera	Total cell number   
(X107)	8.9 (± 1.5)	ns	7.0 (± 1.0)	ns	
	 	IFNgRCNSKO		8.6 (± 2.5)		7.9 (± 2.3)		
	 	WT chimera		10.0 (± 1.7)		6.6 (± 1.8)		
	 	IFNgRperiKO		8.6 (± 1.8)		7.7 (± 2.0)		
	 	IFNgRKO chimera	CD4 
(x106)	 16.3 (± 3.8)	ns	9.1 (± 1.2)	ns	
	 	IFNgRCNSKO		14.0 (± 4.1)		10 (± 2.8)		
	 	Wt chimera		17.7 (± 3.7)		9.9 (± 2.2)		
	 	IFNgRperiKO		17.6 (± 4.4)		13.3 (± 3.2)		
	 	IFNgRKO chimera	CD8 
(X106)	 10.9 (± 2.9)	ns	6.8  (± 1.0)	ns	
	 	IFNgRCNSKO		9.6 (± 2.0)		6.9 (± 2.0)		
	 	WT chimera		11.0 (± 2.0)		6.6 (± 1.2)		
	 	IFNgRperiKO		10.5 (± 2.5)		8.6 (± 1.9)		
	MOG recall responses*	IFNgRKO chimera	Th1
 (x104)	54.8 (± 16.4)	ns	5.0 (± 1.5)	ns	
		IFNgRCNSKO		26.1 (± 8.0)		3.1  (± 0.7)		
		WT chimera		33.6 (± 11.6)		1.9  (± 0.6)		
		IFNgRperiKO		24.8 (± 6.4)		1.8  (± 0.1)		
		IFNgRKO chimera	Th17 
(x104)	46.2 (± 8.8)	ns	5.3 (± 1.5)	ns	
		IFNgRCNSKO		20.5 (± 4.9)		4.7 (± 1.3)		
		WT chimera		25.0 (± 9.7)		2.9 (± 0.7)		
		IFNgRperiKO		15.8 (± 3.6)		3.6 (± 0.3)		
		IFNgRKO chimera	Th1/17 
(x103)	 162.0           (± 62.6)	ns	8.6 (±2.6)	ns	
		IFNgRCNSKO		57.3 (± 19.2)		7.5 (±2.1)		
		WT chimera		114.3            (± 54.2)		3.9 (±1.3)		
		IFNgRperiKO		62.1 (± 18.1)		4.9 (±0.8)		
		IFNgRKO chimera	Tc1 
(x103)	47.1 (± 13.1)	ns	6.8 (± 3.3)	ns	
		IFNgRCNSKO		29.8 (± 6.6)		6.7 (± 2.0)		
		WT chimera		28.1 (± 9.7)		5.0 (±1.7)		
		IFNgRperiKO		41.7 (± 21.2)		4.0 (± 1.1)		
		IFNgRKO chimera	Tc17 
(x103)	28.6 (± 5.4)	ns	9.4 (± 2.2)	ns	
		IFNgRCNSKO		18.4 (± 6.0)		10.2 (± 5.5)		
		WT chimera		24.0 (± 5.1)		11.1 (±5.3)		
		IFNgRperiKO		28.2 (± 10.4)		9.1 (± 3.1)		
		IFNgRKO chimera	Tc1/17 
(x103)	7.7 (± 2.5)	ns	1.5 (± 0.7)	ns	
		IFNgRCNSKO		3.0 (± 1.3)		2.2 (± 0.7)		
		WT chimera		5.4 (± 2.6)		1.0 (± 0.3)		
		IFNgRperiKO		6.7 (± 2.6)		1.8 (± 1.0)		
		IFNgRKO chimera	TNFa+ cells (X105 )	4.6 (± 0.8)	ns	24.1 (± 3.5)	0.053	
		IFNgRCNSKO		3.5 (± 1.2)		12.6 (± 2.5)		
		WT chimera		3.9 (± 0.9)		9.0 (± 2.0)		
		IFNgRperiKO		3.2 (± 0.9)		10.5 (± 1.7)		
		IFNgRKO chimera	IFNg+ cells 
(X105 )	2.6 (± 0.6)	ns	1.5 (± 0.7)	ns	
		IFNgRCNSKO		1.5 (± 0.4)		1.0 (± 0.3)		
		WT chimera		1.8 (± 0.5)		0.5 (± 0.1)		
		IFNgRperiKO		1.4 (± 0.3)		0.6 (± 0.2)		
		IFNgRKO chimera	IL17+ cells 
(X105 )	3.7 (± 1.1)	ns	1.6 (± 0.3)	ns	
		IFNgRCNSKO		2.1 (± 0.5)		1.4 (± 0.4)		
		WT chimera		2.1 (± 0.3)		1.2 (± 0.5)		
		IFNgRperiKO		1.9  (± 0.4)		1.3 (± 0.4)		
		IFNgRKO chimera	IFNg+IL17+ cells (X104 )	7.1 (± 1.9)	ns	1.5 (± 0.3)	0.01	
		IFNgRCNSKO		2.5 (± 0.7)		1.0 (± 0.2)		
		WT chimera		3.9 (± 1.4)		0.5 (± 0.1)		
		IFNgRperiKO		3.2 (± 0.8)		0.9 (± 0.2)		
*absolute numbers obtained after 18hr culture in the presence of MOG35-55


Additional Table 2 (continued)
CNS	 	 	day 14 pi	day 21 pi	
	Group	Cell type	Absolute number	p	Absolute number	p	
	IFNgRKO chimera	 Total 
(X106)	1.8 (± 0.4)	ns	1.6 (± 0.4)	p=0.015	
	IFNgRCNSKO		1.8 (± 0.3)		1.3 (± 0.3)		
	Wt chimera		3.0(± 0.4)		1.8 (± 0.2)		
	IFNgRperiKO		1.9 (± 0.3)		2.6 (± 0.5) *		
	IFNgRKO chimera	CD4 
(X105)	 7.8 (± 2.2)	ns	4.1 (± 0.8)	ns	
	IFNgRCNSKO		 6.6 (± 1.5)		3.2 (± 0.7)		
	WT chimera		  9.1 (± 1.7)		4.4 (± 0.4)		
	IFNgRperiKO		  4.1 (± 0.6)		5.0 (± 0.4)		
	IFNgRKO chimera	CD8 
(X104)	  4.4 (± 1.6)	ns	3.2 (± 0.8)	0.071	
	IFNgRCNSKO		4.4 (± 1.4)		5.5 (± 1.4)		
	WT chimera		 6.2 (± 0.7)		6.0 (± 0.8)		
	IFNgRperiKO		5.6 (± 1.1)		8.9 (± 1.4)		
	IFNgRKO chimera	Th1 
(X103)	  8.0 (± 3.9)	ns	15.2 (± 5.2)	ns	
	IFNgRCNSKO		 8.4 (± 4.2)		7.7 (±2.2)		
	WT chimera		  10.8 (± 2.1)		8.4 (±3.4)		
	IFNgRperiKO		 5.0 (± 1.0)		9.1 (± 2.5)		
	IFNgRKO chimera	Th1/Th17 
(X103)	 2.0 (± 0.6)	ns	2.6 (± 1.1)	ns	
	IFNgRCNSKO		 1.9 (± 0.8)		1.6 (± 0.5)		
	WT chimera		2.5 (± 1.0)		1.3 (± 0.3)		
	IFNgRperiKO		 1.2 (± 0.1)		2.3 (± 0.4)		
	IFNgRKO chimera	Tc1 
(X102)	0.5 (± 0.2)	ns	8.0 (± 3.6)	ns	
	IFNgRCNSKO		 1.3 (± 0.6)		23.2 (± 13.4)		
	Wt chimera		  0.6 (± 0.4)		14.1 (± 6.1)		
	IFNgRperiKO		 1.0 (± 0.7)		8.5 (± 4.0)		
	IFNgRKO chimera	Tc17 
(X102)	  4.0 (± 1.3)	ns	6.8 (± 2.2)	ns	
	IFNgRCNSKO		  3.7 (± 1.9)		3.6 (± 1.5)		
	WT chimera		 3.9 (± 1.2)		7.5 (± 4.5)		
	IFNgRperiKO		  5.4 (± 1.4)		5.4 (± 1.6)		
	IFNgRKO chimera	Tc1/17 
(X10)	 5.3 (± 3.6)	ns	11.5 (± 6.4)	ns	
	IFNgRCNSKO		  6.4 (± 4.0)		19.2 (± 3.6)		
	WT chimera		 4.2 (± 4.2)		16.8 (± 7.3)		
	IFNgRperiKO		  8.1 (± 3.3)		19.3 (± 4.2)		
	IFNgRKO chimera	Treg 
(X104)	4.0 (± 1.2)	ns	3.4 (± 1.3)	ns	
	IFNgRCNSKO		 3.6 (± 1.0)		3.2 (± 0.7)		
	WT chimera		 5.2 (± 0.5)		5.4 (± 1.1)		
	IFNgRperiKO		  4.5 (± 1.2)		5.3 (± 1.3)		
	IFNgRKO chimera	Treg/Th17	  1.0 (± 0.2)	ns	1.2 (± 0.4)	ns	
	IFNgRCNSKO		 0.9 (± 0.3)		1.8 (± 0.5)		
	WT chimera		 1.3 (± 0.3)		2.5 (± 1.0)		
	IFNgRperiKO		  2.4 (± 0.6)		2.5 (± 0.8)		
	IFNgRKO chimera	Microglia 
(X105)	 2.5 (± 0.5)	ns	 3.1 (± 0.4 )	ns	
	IFNgRCNSKO		  2.1 (± 0.7)		 3.1 (± 0.9 )		
	WT chimera		 1.9 (± 0.3)		 2.5 (± 0.4)		
	IFNgRperiKO		  1.9 (± 0.6)		2.3 (± 0.2)		
	IFNgRKO chimera	Dendritic cells 
(X104)	15.2 (± 7.1)	ns	1.2 (± 0.1)	ns	
	IFNgRCNSKO		26.3 (± 6.4)		0.9 (± 0.3) 		
	WT chimera		38.6 (± 6.7)		1.7 (± 0.2) 		
	IFNgRperiKO		19.2 (± 2.2)		1.9 (± 0.2) 		
	IFNgRKO chimera	TNFa+ cells 
(X104 )	4.8 (± 0.6) 	ns	 13.5 (± 5.6)	ns	
	IFNgRCNSKO		4.3 (± 0.7) 		11.0 (± 5.3)		
	WT chimera		6.4 (± 1.3) 		8.3 (± 3.6)		
	IFNgRperiKO		4.3 (± 0.8) 		8.1 (± 3.1)		
	IFNgRKO chimera	IFNg+ cells 
(X104 )	1.5 (± 0.4)	ns	1.6 (± 0.4)	ns	
	IFNgRCNSKO		1.9 (± 0.5)		1.1 (± 0.3) 		
	WT chimera		3.1 (± 0.8)		1.1 (± 0.2) 		
	IFNgRperiKO		1.3 (± 0.2)		1.3 (± 0.3) 		
	IFNgRKO chimera	IL17+ cells (X104 )	2.9 (± 0.3)	ns	3.1 (± 0.6)	ns	
	IFNgRCNSKO		3.5 (± 1.0)		2.4 (± 0.6) 		
	WT chimera		3.6 (± 1.0)		2.4 (± 0.2) 		
	IFNgRperiKO		1.8 (± 0.3)		3.2 (± 0.4) 		
	IFNgRKO chimera	IFNg+ IL17+ cells (X103)	1.1 (± 0.2)	ns	1.0 (± 0.3)	ns	
	IFNgRCNSKO		1.0 (± 0.2)		1.1 (± 0.4) 		
	WT chimera		1.0 (± 0.1)		1.7 (± 0.5) 		
	IFNgRperiKO		1.6 (± 0.4)		1.2 (± 0.2) 		
	IFNgRKO chimera	IL4+ cells per mm2 spinal cord 	4.2 (± 1.5)	ns	7.6 (± 3.4)	ns	
	IFNgRCNSKO		2.0 (± 0.9)		2.0 (± 0.9)		
	WT chimera		2.9 (± 1.6)		2.1 (± 1.8)		
	IFNgRperiKO		2.3 (± 1.5)		2.1 (± 0.9)		
	IFNgRKO chimera	IL4+ cells per mm2 cerebellum	0.25  (± 0.16)	ns	0.4  (± 0.2)	ns	
	IFNgRCNSKO		0.04 (± 0.022)		0.2 (± 0.1) 		
	WT chimera		0.04 (± 0.025)		0.2 (± 0.1) 		
	IFNgRperiKO		0.02 (± 0.022)		0.1  (± 0.04) 		
	IFNgRKO chimera	B (CD45R+) cells per mm2 spinal cord 	8.7 (± 3.17)	ns	25.0 (± 8.1)	0.022	
	IFNgRCNSKO		6.9 (± 0.58)		4.7 (± 1.4) #		
	WT chimera		34.8 (± 14.7)		16.8 (± 4.9)		
	IFNgRperiKO		16 (± 7.8)		59.3 (± 3.9)#		
	IFNgRKO chimera	B (CD45R+) cells per mm2 cerebellum	2.3 (± 1.0)	ns	6.6 (± 3.3)	0.035	
	IFNgRCNSKO		3.1 (± 2.0)		1.1 (± 0.3)# 		
	WT chimera		13.2 (± 10.3)		7.5 (± 3.5) 		
	IFNgRperiKO		1.4 (± 1.0)		47.1 (± 18.0)#		
#values that showed statistical significance
