# Supplementary figures and images for: IFN-gamma signaling in the central nervous system controls the course of experimental autoimmune encephalomyelitis independently of the localization and composition of inflammatory foci
Source: J Neuroinflammation. 2012 Jan 16;9:7. doi: 10.1186/1742-2094-9-7 (PMC3293042; doi:10.1186/1742-2094-9-7)

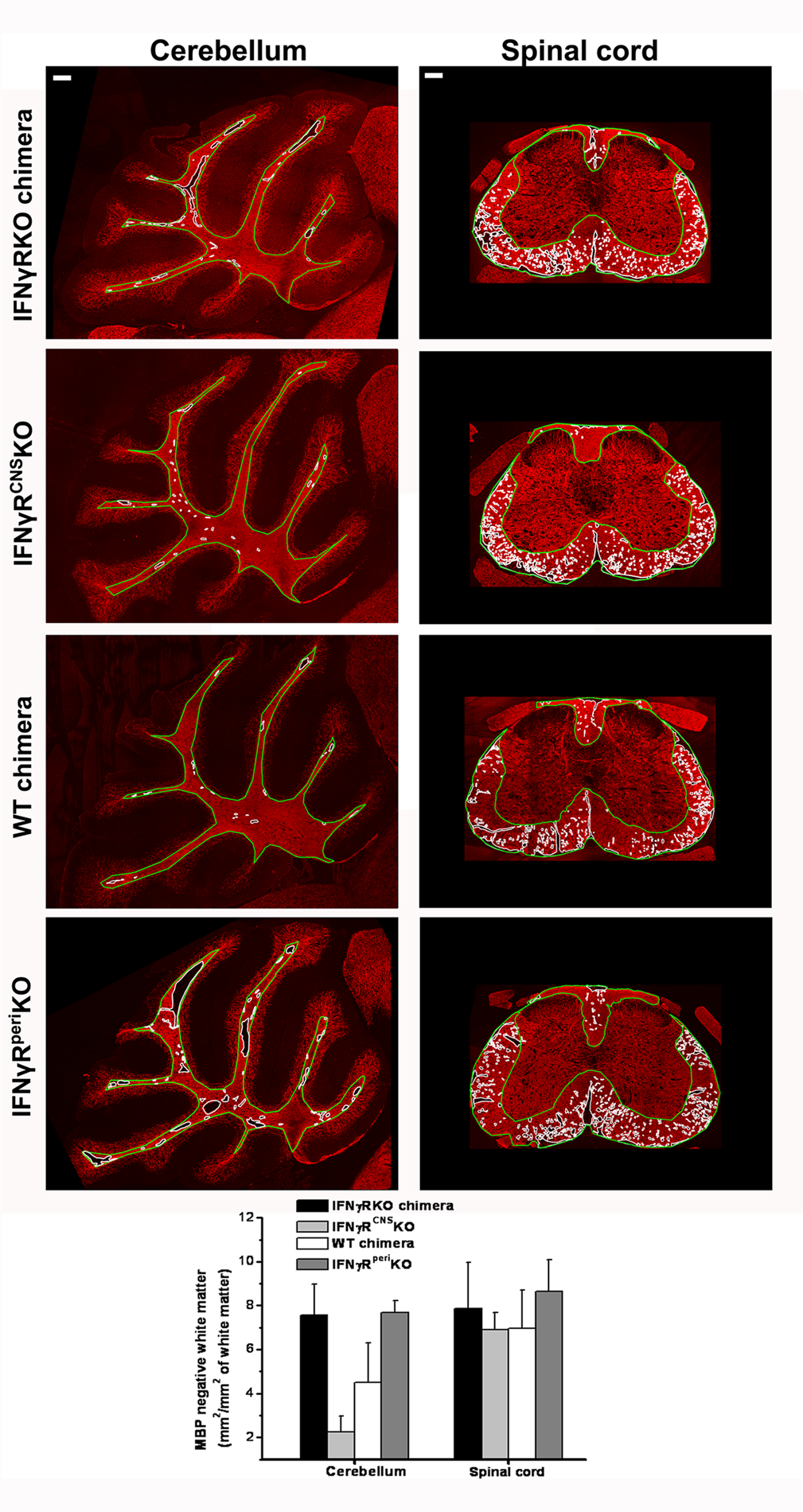

Supplement: Additional file 8 — Loss of myelin does not correlate with the onset of atypical neurological dificits. Fields encompassing the whole spinal cord or cerebellum, isolated from chimeric groups with EAE on day 21 and stained with anti-MBP antibody, were photographed using a 20X objective mounted on a Nikon laser scanning confocal microscope, and images were tiled together using the Nikon NIS-Elements. MBP negative areas were traced and quantified using the ImageJ software. Panels A-H show representative cerebellar and spinal cord sections stained with MBP; areas of demyelination are traced in white and white matter is traced in green. Panel I shows the quantification of MBP-negative areas as mm2/mm2 of white matter. On day 14, IFNγRCNSKO mice showed fewer and smaller demyelinating foci compare to the other groups and this difference approached statistical significance (p = 0.0522). No other statistically significant difference was observed between the groups. [file 1742-2094-9-7-S8.TIFF]
